# Characteristics of Pedaling Muscle Stiffness among Cyclists of Different Performance Levels

**DOI:** 10.3390/medicina57060606

**Published:** 2021-06-11

**Authors:** Isaac López-Laval, Rafel Cirer-Sastre, Francisco Corbi, Sebastian Sitko

**Affiliations:** 1 Faculty of Health and Sport Sciences (FCSD), University of Zaragoza, 22002 Huesca, Spain; sebastiansitko@yahoo.es; 2 Institut Nacional d’Educació Física de Catalunya (INEFC), Universitat de Lleida (UdL), 25192 Lleida, Spain; rcirer@inefc.es (R.C.-S.); f@corbi.neoma.org (F.C.)

**Keywords:** cyclist, myometry, stiffness, incremental cycling test

## Abstract

*Background and Objectives:* The aim of the present study was to compare the impact of an incremental exercise test on muscle stiffness in the rectus femoris (RF), vastus lateralis (VL), biceps femoris (BF), and gastrocnemius (GL) among road cyclists of three performance levels. *Materials and Methods:* The study group consisted of 35 cyclists grouped according to their performance level; elite (*n* = 10; professional license), sub-elite (*n* = 12; amateur license), and recreational (*n* = 13; cyclosportive license). Passive muscle stiffness was assessed using myometry before and after an incremental exercise test. *Results*: There was a significant correlation between time and category in the vastus lateralis with stiffness increases in the sub-elite (*p* = 0.001, Cohen’s *d* = 0.88) and elite groups (*p* = 0.003, Cohen’s *d* = 0.72), but not in the recreational group (*p* = 0.085). Stiffness increased over time in the knee extensors (RF, *p* < 0.001; VL, *p* < 0.001), but no changes were observed in the knee flexors (GL, *p* = 0.63, BF, *p* = 0.052). There were no baseline differences among the categories in any muscle. *Conclusions*: Although the performance level affected VL stiffness after an incremental exercise test, no differences in passive stiffness were observed among the main muscles implicated in pedaling in a resting state. Future research should assess whether this marker could be used to differentiate cyclists of varying fitness levels and its potential applicability for the monitoring of training load.

## 1. Introduction

Road cycling is a popular endurance sport characterized by its cyclic nature, variable intensity, and large training volumes [1]. Among the key determinants of road cycling performance, maximal oxygen uptake (VO_2max_) stands out as one of the values that best represents cardiorespiratory fitness [2]. Furthermore, this parameter has been proposed to classify endurance athletes based on fitness level [3]. Other performance determinants in road cycling include cycling economy or efficiency, tactical and technical skills, psychological resilience, body composition in accordance with the cycling discipline, and muscles’ mechanical properties adapted to the riders’ specialty [2,4]. The muscle mechanical properties might differ among cycling specialties: the most powerful riders are characterized by greater and shorter muscles and a greater predominance of fast twitch fibers when compared to climbers or time-trialists [5]. The relationship between cardiorespiratory fitness and muscle performance factors has been detailed by Hoper et al. (2013), who determined that the percentage of muscle fibers is influenced by the VO_2max_ of the cyclist [6].

Among the mechanical characteristics of the muscle, stiffness provides information regarding its intrinsic property and post-effort response; furthermore, it is one of the main parameters that characterizes the viscoelastic properties of the myofascial complex [7]. A proper conceptualization of muscle stiffness requires an analysis of both muscular architecture and its functional aspect. Concretely, it should be defined as the biomechanical capacity of the tissue (characterized by the type, number, and composition of its muscular fibers) that impedes stretching and distensions [8].

Current literature establishes different conclusions regarding stiffness levels [9,10,11,12]. On the one hand, optimal levels of musculotendinous stiffness are highly correlated to significant increases in muscle performance, especially in situations where the stretch-shorter cycle component is key to optimal performance [9,10,11]. On the other hand, higher stiffness can also be considered a potential threat, since a greater risk of injury has been reported in those athletes who presented greater levels of muscular stiffness as a consequence of high training loads [12]. The literature regarding endurance activities is scarce but has concluded that the reduction in musculotendinous stiffness observed in these types of sports is a consequence of the fatigue generated by submaximal muscle contractions that are sustained in time [13,14]. Related to this, García-Manso et al. (2011) determined that the loss in contractile capacity induced by a long-distance race reflects changes in the neuromuscular response and fluctuations in the contractile capacity of the muscle [13]. Furthermore, Andonian et al. (2016) reported a decrease in quadriceps stiffness caused by an extreme mountain ultra-marathon [14].

Since road cycling is an endurance sport with no impact and in which power is applied to the pedals, the lower limb muscle stiffness of cyclists might differ from other endurance disciplines. To the authors’ knowledge, only four previous studies have analyzed road cycling muscle stiffness with non-invasive tools [15,16,17,18]. Several aspects could be highlighted from these studies: muscular stiffness seems to be an important contributory factor to sprint performance [15] and is proportional to the cyclists’ power output during sprints [16]. Klich et al. (2020) observed higher stiffness in sprinters compared to endurance track cyclists [17]. In this same sense, it is important to highlight the results obtained by Ditroilo et al. (2011), who established that cyclists with higher baseline muscular stiffness suffered greater stiffness losses under fatigue than those with lower baseline levels [18].

The active and passive measurement of muscle stiffness is normally a complex procedure as it requires either muscular biopsies or repeated maximal isometric contractions [8]. Both methods may generate pain and require recovery after the procedure [10]. The utilization of non-invasive techniques for passive measurement of muscle stiffness could be highlighted as a viable alternative, especially when used in a field setting. Tensiomyography, elastography, electromyography, and ultrasounds have been the preferred non-invasive methods in recent years [10], although they still require educated staff and extended time periods for data obtention [8]. The MyotonPRO^®^ (Myoton Ltd., Tallinn, Estonia) is a non-invasive tool that allows for the measurement of passive muscle stiffness through short oscillatory impulses [19] that are generated on the skin and over the area of the analyzed muscle [20]. Previous studies have demonstrated that the device is valid and reliable (ICC = 0.75–0.96; *R*^2^ = 0.95) [8,21] and has been used to measure the main muscles that participate in the pedaling action: rectus femoris (RF), vastus lateralis (VL) [22], hamstring [23], and gastrocnemius (GL) [20]. Klich et al. (2019) proposed myotonometry as an easy and suitable tool to assess the viscoelastic characteristics of muscles in cyclists [24].

It is accepted that both fatigue and performance level of the athlete can influence the changes in muscle stiffness [15,18,20]. Given that previous studies have determined a relationship between stiffness and performance level in other sports, it could be hypothesized that this relationship could also exist in an endurance discipline such as road cycling. However, to date, the differences in muscle stiffness of road cyclists of different performance levels have not been examined. Further, the relationship between initial stiffness level and the response to fatigue after an incremental test is also an area of enquiry that has yet to be investigated. Accordingly, the aim of the present study was to compare the impact of an incremental exercise test on muscle stiffness among road cyclists of three performance levels.

## 2. Materials and Methods

### 2.1. Research Design

**Figure 1:**
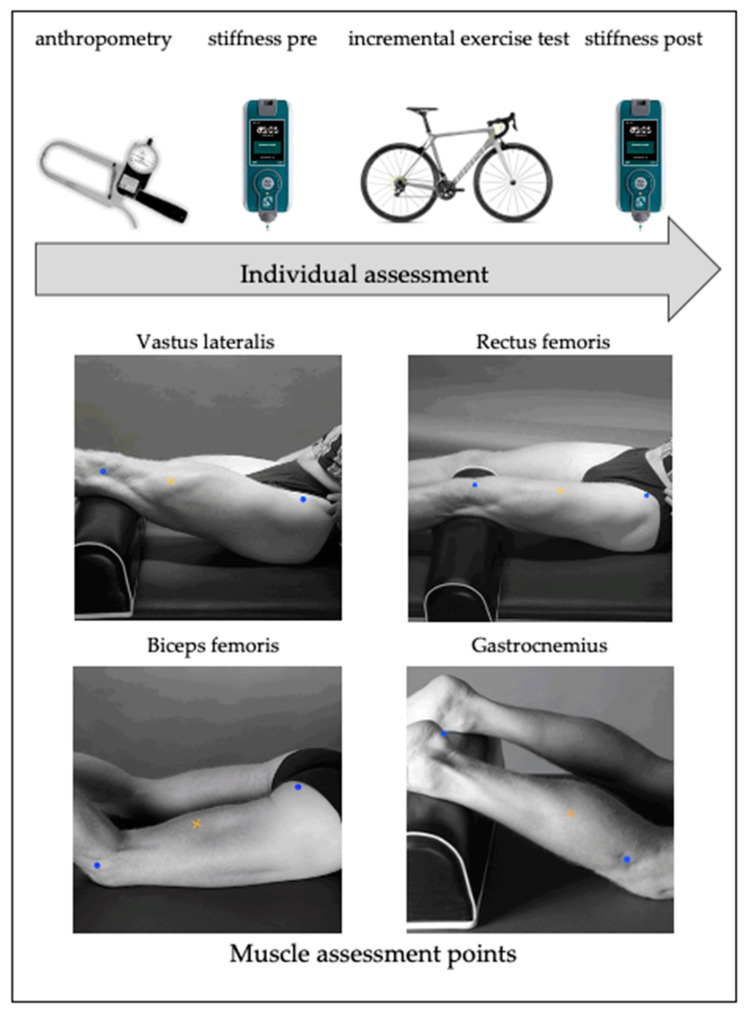
Experimental approach timeline and muscle assessment points. Blue marks: proximal and distal measurement area. Yellow mark: Myoton assessment point.

Thirty-five participants completed this cross-sectional study. The same order was followed during each individual assessment: anthropometry, evaluation of passive muscle stiffness (stiffness pre), incremental exercise test, and assessment of passive muscle stiffness after the incremental exercise test (stiffness post) (see Figure 1). In addition, during the assessment of passive muscle stiffness, the order was always the same between before and after the incremental exercise test and was standardized to avoid the influence of recovery time in muscle stiffness. Measurements were always taken in the same place (University Lab, Río Isuela Sport Center, Huesca, Spain), with a mean temperature of 21 ± 2 °C and mean relative humidity of 52% ± 9%). All participants were assessed during the preparatory phase of the annual training cycle, between the months of September and October and coinciding with the first preparatory meso-cycle. The evaluations were always carried out on Saturdays and Sundays at the same time (between 10 and 12 in the morning) to standardize the measurements and thus organize the schedule of travel to the laboratory. A 48-h rest period was established prior to the initial measurement to guarantee an adequate baseline assessment without fatigue. The assessment of the stiffness was carried out immediately after the completion of the incremental exercise test.

Assessment points placed at VL: 2/3 on the line from the anterior spina iliaca to the lateral side of the patella, RF: 50% on the line from the anterior spina iliaca superior to the superior part of the patella, BF: 50% on the line between the ischial tuberosity and the lateral epicondyle of the tibia, and GL:1/3 of the line between the head of the heel [25].

### 2.2. Participants

G∗Power version 3.1.9.2 [26] was used to estimate the required sample size in a 2 × 3 mixed design for a minimum expected effect size (Cohen’s *F*) of 0.4, an α level of 0.05, and a power (1−β) of 0.95. This procedure returned a minimum number of 30 participants [17]. Thirty-five male cyclists volunteered to participate in the present study. The main characteristics of the participants are reported in Table 1. Participants were allocated to groups according to their performance level: elite, cyclists with a professional license (*n* = 10); sub-elite, cyclists with an U23 or amateur license (*n* = 12); recreational, cyclists that participate in cyclosportive events (*n* = 13). The inclusion criteria were to own a professional, U23, cyclosportive, or masters license. The exclusion criteria were (a) surgical procedures and injuries in the six months prior to the study and (b) use of performance-enhancing drugs in the six months prior to the study. After being informed of the benefits and potential risks of the investigation, all participants signed an informed consent form. The study followed the ethical guidelines of the 2013 Declaration of Helsinki and received approval from the Research Ethics Committee of the autonomous region of Aragon, Spain (the approval code is PI19/447, approved on 4th December 2019).

### 2.3. Data Collection

Participants were weighed and measured by an internationally certified anthropometrist (ISAK level 2). Height (cm) was measured using the SECA-360 measuring rod (SECA^©^, Spain) with a precision of 1 mm, and bodyweight (kg) was measured using scales of the same brand with a precision of 0.1 kg [27]. The tests were carried out between 10 and 12 in the morning. The participants were asked to follow the feeding protocol used for races 3 h before the laboratory appointment. The tests were supervised by a sports doctor and two Bachelor of Science in Physical Activity specialists in performance assessment. The participants performed the entire measurement protocol with their own shorts, slippers, and clipless pedals.

A MyotonPRO^®^ (Myoton Ltd., Estonia) was used to assess the passive stiffness of the main muscles involved in the pedaling action, RF, VL, biceps femoris (BF), and GL, before and after the incremental exercise test. To ensure correct measurements, the assessment points were drawn on the skin following the indications of Hermens et al. [25] (see Figure 1). After removing the tights, and with the cyclist in a lying position on a stretcher, the device was held perpendicular to the skin surface. It was then pushed (0.58 N for 15 ms) against the skin above the muscle area to reach the required depth (*d* = 3 mm). After the red light turned green, five short impulses (tap interval was 0.8 s) were produced automatically by the device in order to induce mechanical oscillations in the soft tissues. In order to guarantee the validity of the data obtained, only those evaluations in which the coefficient of variation was lower than 3% were considered. Otherwise, the assessment was repeated. All measurements were made by the same experienced researcher, and the intra- and inter-rater reliability for this device have been estimated in previous studies. [8,21]. The MyotonPRO^®^ device provides data on the recorded passive muscle stiffness (S, N/m) [28]. The mean values for stiffness were calculated from the responses to the five impulses delivered.

Participants performed an incremental exercise test with gas exchange analysis (CPX/D Med Graphics, St. Paul, MN, USA, EE. UU. Measurement accuracy = 1%) [29] in the laboratory. Cyclists completed the graded exercise tests on their own bikes set up on the Wahoo KICKR Power Trainer (Wahoo Fitness, LLC, Atlanta, Georgia), which allows for power and cadence measurements and has been previously validated [30]. The incremental test was based on the following protocol: 10 min of warm up (5 min 100 W + 5 min 150 W) and increases of 25 W every 3 min [31,32]. The test stopped when a plateau of VO_2_ was reached or, when not seen, at voluntary fatigue when at 100% of estimated HR_max_, a respiratory exchange ratio of ≥1.15 and a rate of perceived exertion (RPE) of ≥18 [32]. The 6–20-point Borg scale was used [33]. All the participants were familiarized with the RPE scale as it was commonly used by their coaches. The scale was shown to the participant in the last 30 s of each one of the steps of the incremental test.

### 2.4. Statistical Analyses

Statistical analyses were performed in R version 4.0.1 (R Core Team 2020) using RStudio (RStudio Team 2020). Variables were visually inspected and described as mean (standard deviation) using the package *rstatix*. Stiffness differences were assessed by fitting an independent linear mixed-effects model for each muscle (rectus femoris, biceps femoris, gastrocnemius lateralis, and vastus lateralis) using the packages *lme4* and *lmerTest*. The models included fixed-effects terms for time (pre and post), category (elite, sub-elite, and recreational), and their interaction. Time at pre and the recreational category were the reference category in each factor, respectively. Random slopes were allowed to vary between moments (time) and random intercepts were allowed to vary among participants (id). Main effects were obtained performing an analysis of variance with each model, and post-hoc pairwise comparisons were performed comparing estimated marginal means using the emmeans package. The effect size of main effects was reported using partial eta squared (η^2^_P_) and interpreted as follows: η^2^_P_ < 0.01 “small”, η^2^_P_ < 0.06 “medium”, η^2^_P_ < 0.14 “large”. Differences in estimated marginal means and their 95% confidence intervals were reported as absolute effect size, and Cohen’s d with Hedges correction and their 95% confidence intervals were reported as standardized effect size. Cohen’s d was interpreted as follows: |*d*| < 0.2 “negligible”, |*d*| < 0.5 “small”, |*d*| < 0.8 “medium”, otherwise “large”. Normality of residuals was assessed using the Shapiro–Wilk test and Q–Q plots, heteroscedasticity was assessed using the Breusch–Pagan test, and model performance was evaluated using Akaike information criterion and R^2^. All assumptions and performance functions were assessed using the package performance. Statistical significance was assumed when *p* < 0.05.

## 3. Results

**Figure 2:**
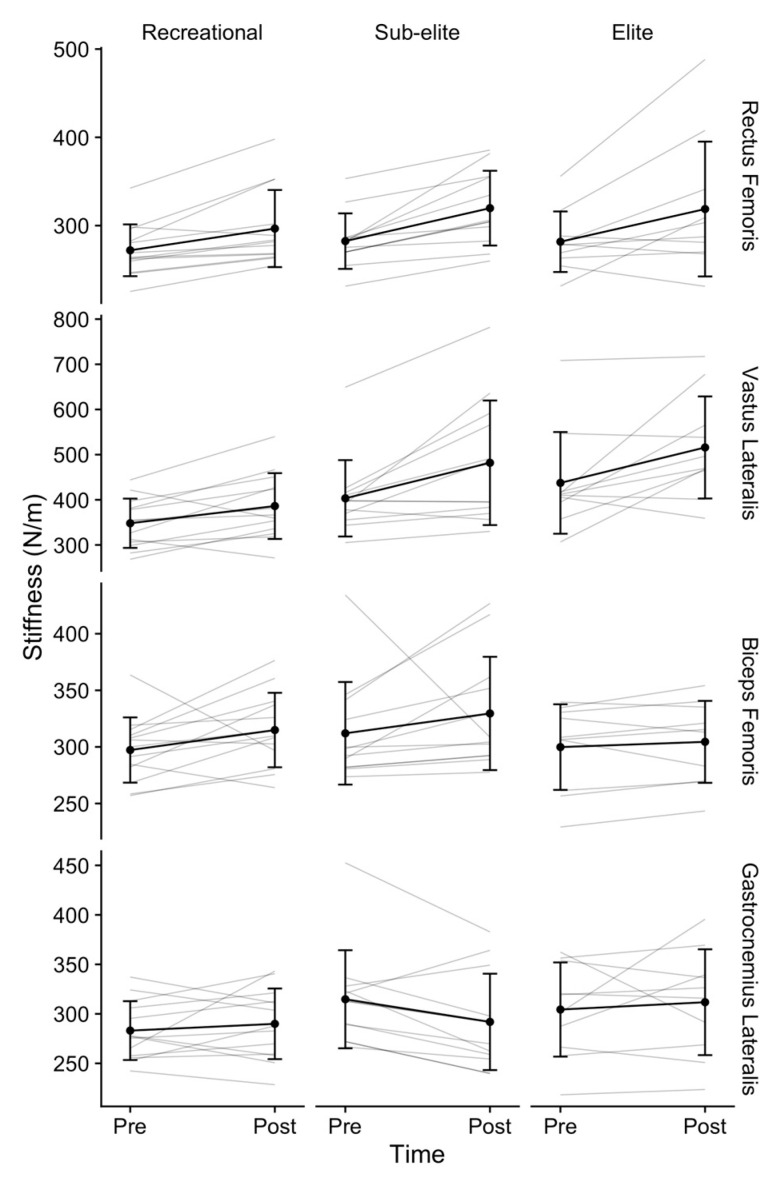
Stiffness by group, muscle, and time. Dots indicate group means whereas vertical error bars indicate standard deviations for each mean.

There was a significant correlation between time and category in the VL with stiffness increases in the sub-elite (*p* = 0.001, Cohen’s *d* = 0.88) and elite groups (*p* = 0.003, Cohen’s *d* = 0.72), but not in the recreational group (*p* = 0.085) (Figure 2). There were no differences among categories in the RF (F(2, 32) = 0.7, *p* = 0.53), GL (F(2, 32) = 0.9, *p* = 0.41), and BF (F(2, 32) = 1, *p* = 0.39). Additionally, baseline stiffness was comparable between categories in all muscles. Stiffness increased over time in both knee extensors, RF (F(1, 32) = 31.9, *p* < 0.001, η^2^_P_ = 0.5) and VL (F(1, 32) = 24.4, *p* < 0.001, η^2^_P_ = 0.2), but no changes were observed in the knee flexors, GL (F(1, 32) = 0.2, *p* = 0.63, η^2^_P_ = 0) and BF (F(1, 32) = 4.1, *p* = 0.052, η^2^_P_ = 0.1).

## 4. Discussion

The purpose of this study was to analyze the passive muscle stiffness of the main muscles involved in the pedaling action in a group of 35 cyclists classified by performance level. Furthermore, the effect of an incremental exercise test until exhaustion on the variations of passive muscle stiffness was also studied to determine whether performance level has an effect on the post-effort muscular response. The main findings of this study could be highlighted as; (i) there were no differences in the resting passive muscle stiffness of the muscles involved in the pedaling action between cyclists categorized by performance level, (ii) an exposition to an incremental exercise test until exhaustion caused an increase in passive muscle stiffness of the knee extensor muscles regardless of the performance group without resulting in modifications of the knee flexor and ankle extensor muscles, and (iii) only the VL differed in its behavior when differentiating elite and sub-elite categories from recreational cyclists. Therefore, it could be determined that there were no significant differences in the passive muscular stiffness analyzed in a resting situation regardless of the level of the cyclist. Furthermore, the subjection to an incremental test until exhaustion only caused an increase in the stiffness of the knee extensors (RF and VL) with significant differences between performance levels (elite and sub-elite vs. recreational) only found for the VL. To the best of the authors’ knowledge, this has been the first study to compare cyclists’ muscle stiffness both in a resting and fatigued situation. In addition, the conclusions reported in this manuscript offer information for cyclists, coaches, and medical staff that help to understand the internal behavior of the muscles and could be considered as a training response variable.

The muscular properties and their behavior during the pedaling action have been studied in the scientific literature [34,35,36]. However, muscle stiffness and especially its variations among cyclists of variable performance levels have been scarcely studied [17,18]. The results obtained in this study differ from those reported by other authors. No significant differences between the analyzed groups (recreational, sub-elite, and elite) or in relation to other parameters such as age, height, or fat percentage in any of the studied muscles (RF, VL, BF, and GL) were obtained. Contrarily, recent studies reported differences in stiffness between performance levels in other sport disciplines. Pruyn et al. analyzed muscle stiffness in netball players classified as elite, sub-elite, and recreational, and reported significant differences between groups (*p* = 0.018). In addition, they concluded by stating that muscular stiffness could be a characteristic that could contribute to a player’s ability to physically perform at an elite level. They also provided an explanation for the high injury rates at elite levels of performance by associating greater levels of stiffness with a higher injury rate [37]. Additionally, Kalkhoven et al. established a relationship between greater muscular stiffness and higher performance in a group of soccer players, highlighting the importance of high stiffness and its contribution to better athletic performance [38].

Regarding cycling, the studies performed by Wastford et al. and Uchiyama et al. should be highlighted. Both determined the importance of high stiffness levels for sprint specialties [15,16]. Uchiyama et al. established a mean value of 186–626 N/m in the VL and determined that this value is proportional to the workload and the power developed by the cyclist [16]. Finally, in the only previous study that analyzed cycling stiffness through myometry, higher values were reported for the knee extensor muscles (VL and RF) in sprinters than in less powerful riders. Our results showed that, in a resting situation, the stiffness characteristics are not associated with a typical endurance performance parameter such as VO_2max_. This finding could be explained because previous studies have analyzed sport disciplines in which the speed component is key to performance [7,37]. This finding does not occur in road cycling, which is a non-impactful discipline characterized by a continuous cyclical movement through coordinated submaximal contractions of the muscles involved in the pedaling action, characteristics that may explain these results. In this study there were no differences in muscle stiffness regardless of the VO_2max_ (348 ± 55 N/m–433 ± 115 N/m). It should be taken into consideration that, despite the lack of statistically significant differences, greater stiffness was observed in those groups with greater aerobic capacity (greater VO_2max_) in the analysis of the VL (recreational; 348 ± 55, sub-elite; 404 ± 85, and elite; 433 ± 115). This increase was not observed for the rest of the analyzed muscles: RF, BF, and GL.

The literature regarding post-effort stiffness in endurance sports is scarce. Both studies by García-Manso et al. [13] and Andonian et al. [14] on long-distance events (Ironman and ultra-marathon, respectively) determined a clear decrease in contractile capacity and a decrease in the stiffness of the quadricep muscles. The results reported in our work showed an increase in the muscle tone of the knee extensors (RF and VL), with significant differences between both muscle groups (*p* < 0.001). Contrarily to what was reported by previous authors, this increase could be explained because the time until the assessment was clearly different in these studies: 4h in the case of the Ironman and days in the case of the ultra-marathon. In our study, muscle stiffness was tested immediately after the incremental test, which may be considered as a relevant factor that may influence the results.

Our results match those obtained by Silva et al., who determined that the RF and VL were the muscles with the highest activation rates during the pedaling action. Regarding the antagonist muscle (BF), a lower but longer total activation was observed [39]. Two years later, the same authors performed a similar analysis, this time with more muscle groups that were analyzed after an incremental test until exhaustion [40]. Again, activation of both RF and VL increased together with some parts of the hamstring muscles (long head of the BF, semitendinosus, and semimembranosus), while there was no activation in the short portion of the BF. The results of our work determined that the BF did not suffer significant differences in stiffness after undergoing a situation of induced fatigue (*p* = 0.052). This could be due to the fact that most of the power during the pedaling action is produced by the RF and VL and to a lesser degree by the BF. This aspect is closely linked to the elevation of post-effort muscle stiffness [15,41]. Additionally, it should be considered that the technique used for the analysis of activation in these studies was EMG and not myometry, an aspect that could influence the results.

In relation to the GL, only three studies have studied the muscular stiffness of this muscle through myometry [20,35,42]. The participation and activation of this muscle in the pedaling action is indisputable, but our results determined that there were no changes in stiffness in the post-effort situation (*p* = 0.63), an aspect that contradicts the results of some studies that highlight the importance of this muscle group involved in flexion and extension of the ankle. Pruyn et al. studied the relationship between muscle stiffness using myometry and variables related to performance in different modalities of team sports and highlighted the importance of enhancing the muscle group composed by the gastrocnemius, soleus, and Achilles tendon in order to achieve success in these sports modalities [42]. Again, the disciplines analyzed to reach this conclusion were based on short, high-intensity motor actions and not cyclical actions composed of submaximal muscle contractions as occurs in a sport such as cycling.

Finally, the correlation between the moment of measurement (pre and post effort) and the study category should be highlighted. In this case, cyclists in the elite and sub-elite groups presented significant differences in the stiffness values of the VL muscle compared to the recreational group. This finding is related to what was reported by Ditroilo et al., who found that cyclists with higher baseline stiffness levels presented greater reductions in muscle stiffness after fatiguing [18]. This would explain why the lower-level group (lower stiffness and lower VO_2max_) presented a behavior that did not match what was seen in the higher-level group. This discrepancy between riders characterized by different fitness levels may suggest that the stiffness control of the knee extensor muscles could be useful as a possible reference for functional tests in the periodic evaluation of cyclists.

Despite the fact that the study sample used in this work covered very high levels of performance, the recreational group presented high basal aerobic levels (VO_2max_ = 49.9 ± 3.1 mL/kg/min), and this could be a limitation that had an impact on the results. In the same way, an incremental test generates maximum aerobic metabolic stimulation but does not induce the same levels of structural fatigue. Future lines of research should not only use groups characterized by lower performance levels but also control groups that would allow a global vision of the muscular stiffness. In addition, the behavior of the muscle should be analyzed in more fatiguing situations such as stage races while, at the same time, considering the specialty of the cyclist. Given that the stiffness values of the VL muscle differed between recreational, elite, and sub-elite cyclists, future research should assess whether this marker could be used to differentiate cyclists of varying fitness levels and its potential applicability for the monitoring of training load.

## 5. Conclusions

The results of this study suggest that there are no differences in the passive muscle stiffness of the muscles involved in the pedaling action between cyclists categorized by performance level. Exposition to an incremental exercise test until exhaustion caused an increase in passive muscle stiffness of the knee extensor muscles, regardless of the performance group, without resulting in modifications of the knee flexor and ankle extensor muscles. Only the VL differed in its behavior when differentiating elite and sub-elite categories from recreational cyclists.

## Figures and Tables

**Table 1:** Summary of the characteristics of the participants.

	Recreational (*n* = 13)	Sub-Elite (*n* = 12)	Elite (*n* = 10)
Age (y)	42.3 ± 7.3	38.2 ± 10.4	32.3 ± 9.2 ^<R (*p* = 0.037)^
Height (cm)	177. 7 ± 8.8	179.25 ± 1.7	177.7 ± 8.3
Mass (kg)	75.7 ± 7.8	74.1 ± 7.7	69.3 ± 8.7
Fat (%)	13.4 ± 4.7	12.8 ± 4.7	8.2 ± 2.1 ^<S (*p* = 0.041); <R (*p* = 0.016)^
VO_2max_ (mL/kg/min)	49.9 ± 3.1	58.6 ± 2.9 ^>R (*p* < 0.001)^	69.6 ± 4.7 ^>S (*p* < 0.001); >R (*p* < 0.001)^
Watts_max_	273.13 ± 2.7	312.5 ± 39.3 ^>R (*p* < 0.001)^	360.0 ± 40.0 ^>S (*p* < 0.001); >R (*p* < 0.001)^

Values are expressed as mean SD; superscripts indicate statistically significant differences and their direction. E = elite, S = sub-elite, R = recreational.
